# Gloucester General Infirmary

**Published:** 1831-11

**Authors:** 


### GLOUCESTER GENERAL INFIRMARY.

### *Peritonitis conjoined with Hernia, but not produced by it.**

Henry Robbins, a stout young man, on the 30th of December, received a kick from a horse, on the right side of the belly: he vomited soon after, and was in constant pain from the time he received the blow to the period when I saw him, which was on the third day. He was bled very largely, (the blood cupped and buffed,) and sent to the hospital. He had stools on the fourth day, on which day, the pain continuing, with occasional vomiting, tension, and tenderness of the abdomen, he was again bled.

On the sixth day his bowels acted well, under purgatives; he had occasional pain only, the tension was as much as ever, but he had not been sick. On this day it was observed that he had a scrotal hernia on the left side, the opposite one to the accident. This, he said, had been there for some time past, but that it disappeared when he lay down. The hernia was now incapable of reduction, and it became a question whether it was not the cause of all the symptoms, rather than the kick. I was called to a consultation.

The tumor was of considerable size; it had descended low in the scrotum, and had a tense feel, was uniformly smooth in its surface, which latter circumstance led me to suspect that it could not be omental. I delivered my opinion against the operation, and in favor of the symptoms being produced by inflammation from the blow, on the following grounds:

1. That the symptoms immediately follow the infliction of the blow.

2. That if the symptoms proceeded from strangulated hernia, the protrusion must be either omentum, or the side only of an intestine; it could not be a circle, because the continuity of the canal was uninterrupted; now the part had not the feel or character of omentum, whilst the size and length of the protrusion prohibited the notion that it was the side only of an intestine.

3. That the firm feel of the protruded part, and the slight tenderness which it possessed, might arise from the peritoneal inflammation, or the effect of the blow having induced the inflammation of the intestines; that this inflammation would naturally affect the portion of the intestine within the sac, inducing a tense tumor, a degree of incarceration from the filling of the protruded part, and consequent tenderness, all of which would subside when the belly subsided, by the removal of the inflammatory affections.

Finally, as the symptoms were, in some measure, subsiding from the means already employed, that they should be continued rather than perform an operation to liberate the intestine, which might be unnecessary; and which, by the infliction of additional violence,

* Fletcher's Med.-Chir. Notes, &c.

would probably destroy the patient, should the existing peritoneal inflammation proceed from the blow of the horse.

From this day (the sixth) he continued to mend; he had no return of sickness, his belly subsided, his skin became cool, his bowels acted, and the tongue was moist. He had been bled, and kept low.

On the fourteenth day, I took the following note: "The patient is convalescent, the tumor is full and prominent, but it no longer offers its former firm and resisting surface. Upon a delicate handling it sinks, and all can plainly hear the gurgling of air passing into the abdomen. But I do not think the intestine returns to the belly, because, though the tumor has obviously diminished, yet a considerable swelling now remains; the diminution of size being the probable effect of the rcmoval of the peritoneal inflammation and consequent stricture upon the gut, and which thus permits its contents to pass upwards into the belly."

"With the view of the case described, I can account for the intestine remaining, by supposing that the peritoneal inflammation, the effect of the blow, had been propagated along that membrane, to the sac of the protruded intestine, between which an adhesion had taken place."

This man was discharged, apparently well; the hernia down; but returned to the hospital on the 2d of February, with his belly swollen, and acutely painful, at times, over its whole surface. At these times, air was felt moving (as he said) along his bowels. There was evidently a fluctuation to be distinguished in the peritoneum. He is now in perfect health, but the rupture still down, irreducibly, which was not the case before the accident.

---

### *Strangulated or Obstructed Scrotal Hernia, produced by an Injury from the Patient's Watch.**

A MAN, about fifty years of age, was brought to the Infirmary, with a strangulated scrotal hernia of four days' standing; his pulse was very low, his belly very tense, obstructed, and tender to the touch; he had hiccough, and a cold clammy sweat pervaded his body.

It was, indeed, an unfavorable case for an operation.

The tumor was not bigger than a small egg, and when the strangulation took place, (in falling,) it appeared that his watch was struck forcibly against it. Was it not the cause of the strangulation or obstruction of the bowels?

In dissecting down to the sac, the external investment of it and the sac itself were so agglutinated together, that no distinction of parts could be made out, and in cutting a portion of the sac upon the director, the intestine itself suffered, which was closely glued to the sac, and doubtless hung with it upon the point of the director.

* Ibid.

From this unavoidable accident taking place, the opening in the gut was, justifiably, considerably enlarged, so as to allow of the escape of the contents of the bowels. The nature of the adhesion of the intestine with the sac was afterwards examined, and found so complete, that the head of the probe could not be insinuated between the two substances.

It was remarked that every touch of the knife, in dissecting down the sac, produced extraordinary bleeding, and doubtless this, as well as the remarkable adhesions between the sac and intestine, were produced by the blow of the watch.

He died on the fourth day from the operation, and I should imagine from the effects of the inflammation on the powers of life, excited by the strangulation, or the blow, before the operation was performed.

All signs of inflammation passed away, as the contents of the abdomen flowed freely through the wound. He appeared to sink like a man exhausted.

It is probable, therefore, that an earlier puncture into the bowel (incapable, from its adhesions, of being otherwise treated) would have saved life, at the expense of an artificial anus.

Or, had the influence of the blow upon the rupture been clearly made out as a probable cause of the strangulation or obstruction, by inducing an inflammatory action of the intestinal canal, and thus increasing the difficulty of the circulation through the hernia, either by the adhesion of the bowel to the sac, (which would limit its own action,) or by filling it with secretion, and thus again by increasing the bulk of the hernia, increasing also the difficulty of returning it into the belly. It is not unlikely that very early and vigorous means, local and general, employed to remove the inflammation, might have saved the patient by preventing its effects; but as the case stood at the time of the patient's admission, it was too late to employ these means, and nothing besides freeing the circulation of the bowels through the hernia, by some operation, could have had a chance of saving life. Had even the one actually practised, though not intended, been done earlier, the chance would have been still greater.

